# Putting PhD students front and center: an empirical analysis using the Effort-Reward Imbalance Model

**DOI:** 10.3389/fpsyg.2023.1298242

**Published:** 2024-01-25

**Authors:** Melanie Vilser, Selina Gentele, Irmgard Mausz

**Affiliations:** ^1^Center for Leadership and People Management, Department of Psychology, Ludwig-Maximilians-University Munich, Munich, Germany; ^2^Department of Business Psychology and HR, International School of Management, Munich, Germany

**Keywords:** Effort-Reward Imbalance Model, overcommitment, PhD students, resilience, stress, well-being, work engagement

## Abstract

**Introduction:**

A doctorate is associated with numerous challenges for many PhD students, including financial insecurities, little support from supervisors, and time pressure. The present study explores well-being of PhD students via the Effort-Reward Imbalance (ERI) model as well as the potential protective factor resilience.

**Method:**

A web-based questionnaire survey was conducted among 1,275 PhD students from Germany. Data was collected at two measurement points over a six-week follow-up period.

**Results:**

As hypothesized, overcommitment was found to mediate the relationship between ERI and perceived stress while no mediation effect was found for work engagement. Resilience strengthened the relationship between ERI and overcommitment, especially for an increasing unfavorable ERI, and counterintuitively did not act as a protective factor.

**Discussion:**

Theoretical and practical implications are discussed, providing a deeper understanding on the ERI model and the negative coping pattern overcommitment in the context of PhD students.

## Introduction

Over the past decades, numerous studies have revealed unfavorable working conditions and high levels of stress for PhD students (Levecque et al., 2017; Sverdlik et al., 2018; Vilser et al., 2022). Hence, PhD students often face precarious working conditions, financial insecurities, lack of time, and challenges in balancing personal and academic commitments (Goller and Harteis, 2014; de Vries, 2020). Moreover, the lack of support from universities, low recognition from supervisors, and heavy workloads exacerbate the serious physical and mental health problems faced by doctoral students (Sverdlik et al., 2018; Hazell et al., 2020). The COVID-19 pandemic has drastically exacerbated the situation for PhD students due to several lockdowns and the increased feeling of loneliness, leading to an upsurge of depression, anxiety, and stress (i.e., Lokhtina et al., 2022; Paucsik et al., 2022; Pyhältö et al., 2023).

To understand the detrimental effects of these stressors on the well-being of PhD students, the Effort-Reward Imbalance (ERI) model provides a valuable framework (Siegrist, 1996). The ERI model states that work-related stress arises from an imbalance between the effort individuals invest in their work and the rewards they receive in return (Siegrist, 2017). Rewards include not only financial aspects but also career prospects and social recognition (Siegrist and Li, 2016). When there is a lack of reciprocity, such as high effort and low reward, individuals experience negative emotions, increased stress, and long-term health consequences (Nguyen Van et al., 2018).

For many doctoral students, a perceived unfavourable ERI may be a critical factor in their well-being. In this context, the intrinsic component of the model, called “overcommitment” emerges as an important factor that plays a significant role as an adverse coping strategy (Siegrist and Li, 2016). Overcommitment refers to an excessive dedication to work, characterized by a willingness to work beyond expectations and difficulties in detaching oneself from job demands (Siegrist and Li, 2020; Kunz et al., 2021). Doctoral students, in particular, may be prone to overcommitment while facing high work demands and low rewards to continue pursuing their goal of earning a doctorate.

In addition to elucidating the process that leads to decreased well-being, it is necessary to understand how individual vs. organizational factors contribute to the well-being of doctoral students. In the following, we focus on the individual perspective, as we aim to investigate how individual characteristics influence the ability to cope with ERI. In recent years, the concept of resilience has gained attention as a potential protective factor in mitigating the negative effects of stress and adversity (Kearns et al., 2008; McCray and Joseph-Richard, 2020; Anders et al., 2022). Resilience is characterized as the process of adapting well in the face of adversity and recovering from difficult experiences (American Psychological Association, 2020). Not only is resilience an inherent trait but also a behavioral characteristic that can be developed through personal resources (Sinclair et al., 2016; Booth and Neill, 2017). In the context of doctoral students, resilience can be described as the acquisition of skills that enable students to cope with the challenges of their doctoral journey while maintaining a sense of assertiveness, confidence, and persistence (Mowbray and Halse, 2010).

With the present study, we contribute to research and practice in several ways. First, little scientific research exists analyzing the well-being of PhD students. Especially by applying the ERI model, we will get valuable insights on the origin of reduced well-being in PhD students. This will for instance make it possible to give recommendations to universities and supervisors of PhD students on how to prevent ERI. Second, in this study, we not only look at the connection between ERI and well-being in PhD students, but also explain the mechanism of deterioration in well-being. Third, with the investigation of the protective factor resilience, we aim to give valuable hints on how to diminish the consequences of ERI in PhD students. By investigating resilience, we will be able to draw practical implications on how to foster resilience in PhD students to help them deal with ERI.

## Theoretical background

This study is based on the Effort-Reward Imbalance Model (Siegrist, 1996), which has been frequently used to address the imbalance between effort put into work and reward received, as well as its effects on health and well-being. The key assumption of the ERI model is that work-related stress is triggered by a lack of social reciprocity between effort and reward. Thus, employees expect to receive adequate rewards from their employer, including “salary or wage (financial reward), career promotion or job security (status-related reward), and esteem or recognition (socio-emotional reward)” in return for performed work (Siegrist, 2017, p. 25). On the contrary, a lack of reciprocity, such as high effort and low reward, leads to negative emotions, increased stress, and ultimately long-term negative health effects (Siegrist and Li, 2016; Nguyen Van et al., 2018). The ERI model is a well-established framework for understanding work-related stress and its implications on people’s health, especially in the context of school and university settings (e.g., Li et al., 2010; Wege et al., 2017; Kunz et al., 2021). It has been extensively tested and validated over the years, with numerous studies confirming its utility in explaining work-related stress and health outcomes (e.g., Kunz, 2019).

Siegrist (2015) identified three specific conditions in which people are willing to persist in high-effort/low-reward work situations while remaining highly committed, namely dependency (having no other choice, e.g., due to advanced age or low skills and knowledge), strategic reasons (e.g., prospect of a favorable career development) and overcommitment. Overcommitment, the intrinsic component of the ERI model, is defined as “a cognitive-motivational pattern of coping with demands characterized by excessive work-related striving” (Siegrist and Li, 2020, p. 7). Overcommitted employees are more likely to engage in additional demands at work, are extremely dedicated, and tend to work more than expected. The three conditions described can easily be applied to the majority of doctoral students, as they are bound to the 3 to 5-year PhD program and often choose it for strategic reasons. Furthermore, Hamilton (2019) found that academic employees have comparatively high scores in overcommitment, while a study from Kearns et al. (2008) lists overcommitment as one of the negative coping patterns applied by doctoral students. In addition, a general imbalance between effort and reward is to be expected during a doctoral program, as doctoral students face numerous challenges and often receive little support and recognition from faculty and supervisors (Beasy et al., 2021). This is supported by Kunz et al. (2021), indicating an unfavorable ERI for PhD students.

Over the last decades, the validity of the ERI model has been investigated by several empirical studies as well as meta-analyses and reviews (e.g., Dragano et al., 2017). Here, the focus was on testing the three central hypotheses formulated by Siegrist, predicting direct effects of each of the three scales: effort, reward, and overcommitment on health. Furthermore, the so-called effort-reward ratio (ERI ratio), quantifying the relationship between effort and reward, should have the strongest effect on health, while overcommitment is expected to moderate the relationship between effort, reward, ERI ratio, and health (Siegrist and Li, 2016). To comprehensively frame the theoretical and empirical foundation, it is worth acknowledging at this juncture that overcommitment may exacerbate the associations between ERI and health outcomes (Feuerhahn et al., 2012). However, the interaction hypothesis, containing overcommitment as the moderator and the ERI ratio as a predictor, could not be supported in several studies (Siegrist and Li, 2016). Rather, current research suggests that overcommitment, described as an individual coping pattern, might act as a mediator instead of a moderator (e.g., Theorell, 2017; Hinsch et al., 2019; Hodge et al., 2020). However, the role of overcommitment as a mediator in the ERI model remains an important area of research, specifically to explain the process that links ERI and decreased well-being (Theorell, 2017; Hinsch et al., 2019; Hodge et al., 2020; Vilser, 2021).

The mediator function is also described by Hinsch et al. (2019, p. 564) referring to Siegrist and Marmot (2004) and the psychological recovery resources by Sonnentag and Fritz (2015). Accordingly, overcommitment “can be understood […] as a reactive behavior aimed at overcoming certain stressors experienced by the individual. […] [This is supported by studies showing that] psychological detachment is meditating associations between job stressors and well-being” (Hinsch et al., 2019, p. 564). This assumption is further underpinned by Kunz et al. (2021), demonstrating the conceptual proximity between the ERI model and the stressor-detachment model. In this model, Sonnentag and Fritz (2015) describe psychological detachment as a mediator between job stressors, such as high workload, and employee well-being and strain. Psychological detachment is characterized by the ability to “mentally disengage from one’s job while being away from work” (Sonnentag and Fritz, 2015, p. 72). Overcommitment in the ERI model describes an excessive commitment to work, which is connected to the willingness to work more than expected as well as the inability to distance oneself from job demands (Siegrist and Li, 2020; Kunz et al., 2021). In this study, the assumption is made that psychological detachment and overcommitment are comparable constructs. Furthermore, the assumption of a mediating effect of overcommitment is adapted.

Looking at the adverse working conditions, high pressure, and several challenges (e.g., publishing papers, raising research funding) within the doctorate, it brings into question how PhD students can handle the numerous hazards. One common protective factor discussed is resilience (Kearns et al., 2008; McCray and Joseph-Richard, 2020; Anders et al., 2022). Resilience results from an interplay of innate abilities and personal resources as well as learnable skills, including a variety of coping strategies, and other protective factors in the environment, and helps to adapt in the face of difficult experiences (American Psychological Association, 2020). Therefore, it is not only a rigid trait of individuals but also a behavioral characteristic that can be acquired by anyone (Sinclair et al., 2016; Booth and Neill, 2017). Resilience in PhD students can be described as the “acquisition of skills that enable students to become more assertive, confident, resilient, persistent and resolute in determining how to progress their PhD while balancing their other commitments” (Mowbray and Halse, 2010, p. 657). Thus, this study regards resilience as the result of successfully applied coping strategies, helping to recover from adversity and grow from it (Booth and Neill, 2017). Further, we assume that resilience acts as a moderator, between the stressor, the subjectively experienced and unfavorable ERI, and the intrinsic component overcommitment. It is expected that high psychological resilience is associated with the ability to apply favorable coping strategies when confronted with certain stressors. This is supported by the fact that although resilience and coping strategies continue to be seen as distinct constructs, current literature suggests that they are interdependent (Rice and Liu, 2016). Thus, Heckenberg et al. (2019) showed that an online mindfulness-based stress reduction program successfully reduced overcommitment by developing various coping strategies such as meditation or yoga. Many other studies have addressed the importance of developing appropriate coping strategies to successfully reduce overcommitment and ERI at work and ultimately improve employee health (Unterbrink et al., 2012; Li et al., 2017). Thus, the study of this issue is relevant because overcommitment is associated with both vital exhaustion and negative physical health outcomes (Siegrist and Li, 2016).

This study examines the experiences of PhD students in the light of the Effort-Reward Imbalance model. While many PhD students show great intrinsic motivation and enthusiasm for their doctoral studies (Guerin et al., 2015; Sverdlik et al., 2018), they also face numerous challenges, ranging from precarious working conditions, financial insecurities, poor work-life balance, and high levels of stress (Goller and Harteis, 2014; Levecque et al., 2017; Sverdlik et al., 2018; de Vries, 2020). As indicated by Stubb et al. (2011), the source of stress of burnout is “[...] not simply an individual symptom, but instead a mismatch in the relationship between the individual and the environment [...]” (p. 34). Thus, it is conceivable that all these circumstances pave the way for an unfavorable ERI within PhD students, as also indicated in a recent study of PhD students by Kunz et al. (2021). Further, it has been shown that overcommitment is prevalent among doctoral students (Kearns et al., 2008; Hamilton, 2019).

To investigate the possible effects of an ERI imbalance on PhD students, the two outcome variables perceived stress and work engagement are chosen in this study. While experiencing stress at work is nearly ubiquitous, PhD students are especially endangered due to the unique working conditions as explained above. Further, prolonged periods of stress lead to physical and mental illnesses, including headaches, colds, back pain, sleep disorders as well as depression, and burnout (Techniker Krankenkasse, 2021). Moreover, work engagement, a construct stemming from positive psychology, and defined as “a positive, fulfilling, work-related state of mind that is characterized by vigor, dedication, and absorption” (Bakker and Demerouti, 2008, p. 209), is analyzed as organizational outcome variable. Hence, work engagement in employees is connected to high levels of energy (vigor), enthusiasm and happiness (dedication) as well as full immersion into work (absorption) (Bakker and Demerouti, 2008), often shown in PhD students (Guerin et al., 2015; Sverdlik et al., 2018).

**Figure 1:**
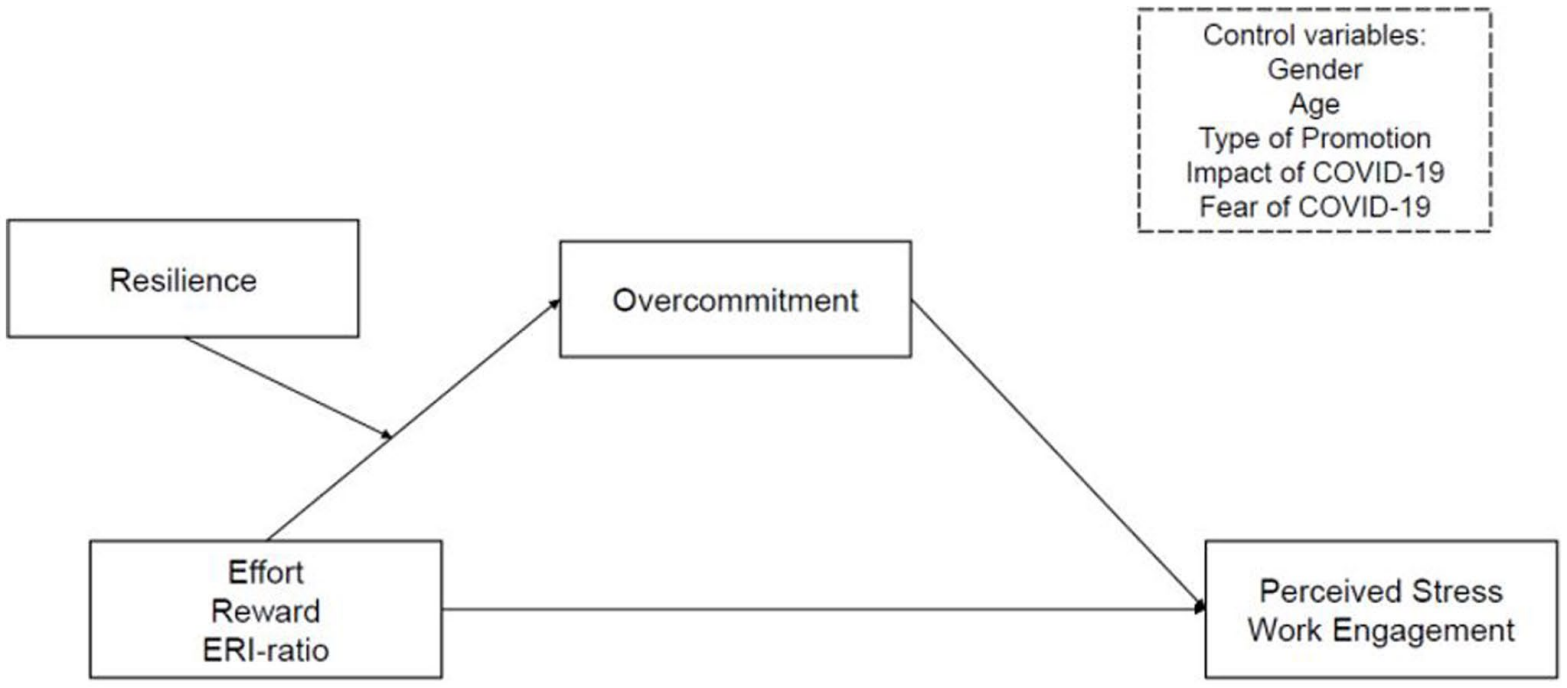
Research model of the study.

In accordance with the reasoning above, we hypothesize that high effort is positively associated with perceived stress and negatively associated with work engagement (1a) and low reward (1b) as well as high overcommitment (1c) is positively associated with perceived stress and negatively associated with work engagement. Subsequently, we expect that the combined measure quantifying the imbalance between high effort and low reward (ERI ratio) is positively associated with perceived stress and negatively associated with work engagement, exceeding the effect sizes produced by the single scales (2). Further, we assume that overcommitment mediates the positive relationship between effort-reward imbalance and perceived stress (3a) and the negative relationship between effort-reward imbalance and work engagement (3b). Also, we hypothesize that the mediation between effort-reward imbalance, via overcommitment, on perceived stress (4a) and work engagement (4b) is moderated by resilience. All hypotheses were pre-registered (Gentele et al., 2022; Figure 1).

## Methods

### Sample

The data for this study was collected through online questionnaires at two measurement points with a six-week interval, ranging from April 2022 to June 2022 and thus during the COVID-19 pandemic. To assemble our study cohort, we initiated contact with all 156 German universities offering PhD programs, as listed in Hochschulkompass 2022, alongside the 13 primary scholarship providers affiliated with the German Federal Ministry of Education and Research. Of these, 100 universities and six scholarship providers agreed to share the study. To create higher visibility, the study was also disseminated via LinkedIn and snowball systems in the author’s direct environment. In general, no other rationale was applied for finding participants for this study than being an active PhD student in Germany.

In total, 1,275 PhD students completed the survey during the first measurement point while 705 people did so during the second (dropout rate: 54.48%). The primary characteristics of the study sample are displayed in Table 1.

**Table 1:** Sociodemographic and PhD-related characteristics of participants.

	First measurement point		Second measurement point	
	*n*	%	*n*	%
Age	30.44 (5.98)		30.10 (6.11)	
Gender				
Male	445	34.9%	229	32.4%
Female	813	63.8%	473	67.0%
Non-binary	11	0.9%	3	0.4%
Field of research^1^				
Mathematics and natural science	395	31.0%	219	31.0%
Law, economics, social sciences	261	20.5%	137	19.4%
Humanities	224	17.6%	131	18.6%
Engineering	157	12.3%	84	11.9%
Human medicine, health science	77	6.0%	49	6.9%
Sports	32	2.5%	13	1.8%
Agricultural, forestry, nutrition	23	1.8%	13	1.8%
Veterinary medicine	16	1.3%	12	1.7%
Art	21	1.6%	10	1.4%
Others	46	3.6%	27	3.8%

^1^23 People did not reply regarding their study subject.

### Measurements

Below, the measures and control variables relevant to this paper are presented. For all scales, doctoral students were asked to refer only to their doctorate and not to other jobs when rating the statements. The survey was conducted in German.

#### Effort-Reward Imbalance

To measure the experienced Effort-Reward Imbalance of PhD students, the ERI questionnaire for doctoral students was used (ERI-PhD, Vilser et al., 2024). The questionnaire includes 18 items, with six items capturing the subscale effort (*α* = 0.78; *ω* = 0.78), eight items the subscale reward (*α* = 0.77; *ω* = 0.75) and four items the subscale overcommitment (*α* = 0.83; *ω* = 0.83). Therefore, the reliability of the three subscales can be considered high for overcommitment and acceptable for reward and effort (Blanz, 2015). The items were rated on a 4-point Likert scale (response scales ranged from 1 = *strongly disagree* to 4 = *strongly agree*). Regarding the effort subscale, the PhD students were asked, for example, whether they have constant time pressure due to the heavy workload in their PhD. Furthermore, the reward subscale included for example the item “I receive the respect I deserve from my supervisors or a respective relevant person,” while “As soon as I get up in the morning, I start thinking about problems related to my PhD” was a sample item of the subscale overcommitment. The ERI ratio was calculated according to Siegrist’s formula: effort/reward × correction factor (0.75). While an ERI ratio < 1 indicates favorable conditions with high reward and low effort, an ERI ratio > 1 indicates unfavorable conditions with high effort and low reward (Siegrist, 2002).

#### Work engagement

Work engagement was measured using the German short version of the Utrecht Work Engagement Scale with 9 items (UWES-9; Schaufeli et al., 2006) including the three subscales vigor (*α* = 0.87; *ω* = 0.87), dedication (*α* = 0.86; *ω* = 0.86) and absorption (*α* = 0.86; *ω* = 0.86). Cronbach’s alpha for the entire scale, including all 9 items, was *α* = 0.95. Thus, the internal consistency for all three subscales and the scale can be considered high (Blanz, 2015). Participants rated their work engagement on a 7-point Likert scale ranging from 1 (*never*) to 7 (*every day*). A sample item for the subscale vigor was: “At my work, I feel bursting with energy.” The subscale dedication was measured, for example, by asking the PhD students to what extent their job inspires them and the subscale absorption included the item “I feel happy when I am working intensely.”

#### Perceived stress

To assess the subjective stress perception of PhD students, the German short version of the Perceived Stress Scale (PSS-4) with 4 items was used (Schneider et al., 2020). The items were rated on a 5-point Likert scale ranging from 1 = *never* to 5 = *very often*. A sample item was: “In the last month, how often have you felt difficulties were piling up so high that you could not overcome them?”. The reliability of this scale using Cronbach’s Alpha and McDonald’s Omega was acceptable (*α* = 0.79; *ω* = 0.79).

#### Resilience

Resilience in PhD students was measured using the German Brief Resilience Scale with 6 items (Chmitorz et al., 2018). Participants were asked to rate the statements on a 5-point Likert scale ranging from 1 (*strongly disagree*) to 5 (*strongly agree*). A sample item of this scale was: “I tend to bounce back quickly after hard times.” The reliability of this scale was high with a Cronbach’s Alpha of *α* = 0.83; and a McDonald’s Omega of *ω* = 0.83.

#### Control variables

Five control variables were included in this study. First, the sociodemographic control variables age and gender were included as these could possibly impact the doctoral experience (Kurtz-Costes et al., 2006). Thus, age was found to influence work engagement, as older workers have more resources available to better cope with workplace stressors (Kim and Kang, 2017). Furthermore, the type of promotion was included to account for the different initial situations of the various promotion models (e.g., structured doctoral program, research assistant at university). Finally, two items were added to control for the impact and fear of COVID-19, as research work had to be interrupted, conferences canceled and contact restrictions had to be maintained during the pandemic (Anders et al., 2022).

Thus, the PhD students were asked to rate the impact of COVID-19 on their PhD project on a 7-point Likert scale (ranging from 1 = *strongly disagree* to 7 = *strongly agree*). The item “The COVID-19 pandemic has a negative impact on my PhD project” was formulated based on Van Der Feltz-Cornelis et al. (2020) and adapted to the PhD context.

Lastly, fear from COVID-19 was asked, using one item of the Fear of COVID-19 Scale (Fatfouta and Rogoza, 2021). There, participants were asked to rate their fear of COVID-19 on a 7-point Likert scale (ranging from 1 = *strongly disagree* to 7 = *strongly agree*).

### Data analysis

Before analyzing the data, the revised items were recoded – specifically, 5 items of the ERI scale, 2 items of the Perceived Stress scale, and 3 items of the Resilience scale. Subsequently, the mean score for each scale mentioned above was calculated. Additionally, the sum score of the ERI scales and the ERI ratio were computed using Siegrist’s formula (effort/reward × correction factor). Following this, we tested the requirements of our statistical analysis.

Two Confirmatory Factor Analyses (CFA) were conducted using Jamovi to examine the construct validity of the ERI questionnaire as well as the distinctiveness of the constructs overcommitment and work engagement due to their similarity in terms of content. Additionally, the statistic software IBM SPSS 25 was used to test the predicted hypotheses. For hypotheses 1a, 1b, and 1c, two separate multiple regression analyses were conducted using the single scales effort, reward, and overcommitment as independent variables (predictors). Perceived stress and work engagement acted as the two dependent variables. Furthermore, two separate linear regression analyses were performed to test hypothesis 2, with ERI ratio as an independent variable and perceived stress and work engagement as the outcome variables. The adjusted R-squared was used to assess the goodness-of-fit measure (Gordon, 2023).

Hypotheses 3a and 3b were tested via two mediation analyses using model 4 of the PROCESS macro for SPSS (Hayes, 2022). Here, the ERI ratio was the predictor, overcommitment the mediator and work engagement as well as perceived stress the dependent variables. For the analyses, PROCESS macro uses ordinary least square regressions. Moreover, the number of bootstrap samples was set to 5,000, the level of confidence for all confidence intervals was 95% and a heteroscedasticity consistent standard error was applied (Hayes, 2022).

Two moderated mediation analyses were conducted to test hypotheses 4a and 4b, using model 7 of the PROCESS macro for SPSS (Hayes, 2022). The level of confidence intervals was set to 95,000 and the number of bootstrap samples to 5,000. The ERI ratio was used as a predictor variable, overcommitment as the mediator and work engagement as well as perceived stress as the dependent variables. Additionally, resilience functioned as the moderator. Further we adjusted the settings of the model, so that the outcome included the data for visualizing the conditional effect of the focal predictor via simple slope (for details see step by step guide from Hayes, 2022).

## Results

### Confirmatory factor analyses

Below, the results of the CFA are displayed, testing the construct validity of the ERI questionnaire as well as the distinctiveness of overcommitment and work engagement.

#### Construct validity of ERI-questionnaire

To test the construct validity of the ERI Questionnaire, a CFA was performed using Jamovi (2021). Looking at the model fit indices, the results showed an acceptable fit [χ^2^ (*N* = 1.275, *df* = 132) = 1,154, *CFI* = 0.861, *TLI* = 0.838, *RMSEA* = 0.078] according to Beauducel and Wittmann (2005) suggesting a CFI ≥ 0.90 and Hu and Bentler (1999) suggesting a RMSEA ≤0.08.

#### Distinctiveness of overcommitment and work engagement

To test for the distinctiveness of the constructs overcommitment and work engagement, due to their conceptual similarity, a second CFA was performed. Two nested models were tested against each other: a one-factor model combining the two constructs and a two-factor model differentiating between the two constructs. The results are displayed in Table 2, showing a better fit for the two-factor model based on the fit indices (Beauducel and Wittmann, 2005). It should be noted that the chi-square test was significant for both models, indicating poor fit. However, because the chi-square test is sensitive to large sample sizes, additional fit indices were used to evaluate the fit, indicating a better fit of the two-factor model (Hooper et al., 2008).

**Table 2:** Results from the CFA testing the distinctiveness of overcommitment and work engagement.

Model	*df*	*χ^2^*	CFI	TLI	RMSEA
One-factor model	65	2205***	0.806	0.768	0.161
Two-factor model	64	335***	0.975	0.970	0.052

*N* = 1,275. CFI, Comparative Fit Index; TLI, Tucker-Lewis Index; RMSEA, Root Mean Square Error of Approximation. ^***^p < 0.001.

### Descriptive statistics and correlations of variables

The correlations, means and standard deviations of all variables are shown in Table 3.

**Table 3:** Means, standard deviations, and correlations as well as asymmetry and kurtosis of main variables.

Variable	*M*	*SD*	1	2	3	4	5	6	Asymmetry	Kurtosis
1. Effort	2.62	0.61	–	–					−0.06	−0.42
2. Reward	2.74	0.52	–	−0.35**	–				−0.23	−0.21
3. Over-commitment	2.76	0.72	–	0.56**	−0.28**	–			−0.34	−0.47
4. Resilience	3.26	0.76	–	−0.10**	0.17**	−0.25**	–		−0.17	−0.38
5. Perceived Stress	2.80	0.77	–	0.31**	−0.44**	0.49**	−0.38**	–	0.08	−0.44
6. Work Engagement	4.47	1.17	–	0.03	0.33**	−0.11**	0.20**	−0.35**	−0.17	−0.41

*N* = 1,275. ^**^p < 0.01.

### Hypotheses testing

The results of the regression, the mediation, and the moderated mediation analyses are presented below. After including the previously defined control variables in the model, no changes in the pattern of results were observed. Thus, non-controlled results are reported. However, it is worth mentioning that the negative impact of COVID-19 was positively associated with perceived stress for all analyses, while age was negatively associated with perceived stress for all regression analyses. For the outcome variable work engagement, COVID-19 was found to be negatively associated in all regression analyses, while age was positively associated with work engagement only in the multiple regression analyses including the single scales effort, reward, and overcommitment.

#### Regression analyses

Two separate multiple regression analyses were conducted to test the relationship between effort (H1a), reward (H1b), and overcommitment (H1c) and the dependent variables perceived stress and work engagement, respectively. For perceived stress, the overall regression model was significant [*F*(3, 1,265) = 160.800, *p* < 0.001]. Furthermore, 27.4% of the variance of perceived stress could be explained by the model (*R*^2^_adjusted_ = 0.274). Regarding the three predictors (effort, reward, and overcommitment), all predictors had a significant relationship with perceived stress with overcommitment having the biggest effect size (see Table 4). Thus, reward was negatively associated with perceived stress, while overcommitment and effort were positively associated with perceived stress.

**Table 4:** Regression analysis for the prediction of the dependent variable perceived stress.

Predictor	*b*	*SE b*	*β*	*t*	*p*	*r*_partial_
1. Effort	0.139	0.038	0.110	3.709	<0.001	0.104
2. Reward	−0.389	0.038	−0.260	−10.126	<0.001	−0.274
3. Overcommitment	0.332	0.031	0.307	10.585	<0.001	0.285

*N* = 1,275.

Looking at the results for the outcome variable work engagement, the overall regression model was significant [*F*(3, 1,265) = 61.674, *p* < 0.001], being able to explain 10.5% of the variance of work engagement (*R*^2^_adjusted_ = 0.105). All predictors showed a significant relationship with work engagement (see Table 5). Thereby, reward had the biggest effect on work engagement, followed by overcommitment and effort. Consequently, effort and reward were positively associated with work engagement, while overcommitment was negatively associated with work engagement.

**Table 5:** Regression analysis for the prediction of the dependent variable work engagement.

Predictor	*b*	*SE b*	*β*	*t*	*p*	*r*_partial_
1. Effort	0.286	0.063	0.150	4.544	<0.001	0.127
2. Reward	0.745	0.064	0.330	11.583	<0.001	0.310
3. Overcommitment	−0.145	0.053	−0.089	−2.762	0.006	−0.077

*N* = 1,275.

Moreover, two separate linear regression analyses were performed to test the relationship between ERI ratio and the two outcome variables perceived stress and work engagement, respectively. Also, it was determined whether the ERI ratio exceeds the effect sizes produced by the single scales on the outcome variables (H2). For perceived stress, the overall regression model was significant [*F*(1, 1,267) = 146.261, *p* < 0.001], being able to explain 19,1% of the variance of perceived stress (*R*^2^_adjusted_ = 0.191). Further, ERI ratio showed a significant positive relationship with perceived stress (*β* = 0.438, *p* < 0.001; *r_partial_* = 0.438). Comparing the adjusted R-squared, the model including all three predictors showed a higher goodness-of-fit than the model including ERI ratio only. Thus, hypothesis 2 could only partially be supported for the outcome variable perceived stress.

For the outcome variable work engagement, the overall regression model was significant [*F*(1, 1,267) = 57.631, *p* < 0.001], being able to explain 3.3% of the variance of work engagement (*R*^2^ = 0.033). Further, ERI ratio showed a significant negative relationship with work engagement (*β* = −0.182, *p* < 0.001, *r_partial_* = −0.182). As for the outcome variable perceived stress, hypothesis 2 could only be partially supported, as the goodness-of-fit, assessed by the adjusted R-squared was higher for the regression model including all three predictors than ERI ratio only.

#### Mediation analyses

Two separate mediation analyses were conducted to test whether overcommitment mediates the relationship between the ERI ratio and the two dependent variables, perceived stress (H3a) and work engagement (H3b). Regarding hypothesis 3a, using perceived stress as the dependent variable, a total effect of ERI ratio on perceived stress was found (*β* = 0.5216, *p* < 0.001). Furthermore, ERI ratio significantly predicted overcommitment (*β* = 0.8941, *p* < 0.001), which in turn predicted perceived stress (*β* = 0.3004, *p* < 0.001). Thus, hypothesis 3a was supported, showing that overcommitment mediates the relationship between ERI ratio and perceived stress. All results for the mediation analysis using perceived stress as the outcome variable can be found in Table 6.

**Table 6:** Direct and indirect effects of ERI ratio on perceived stress mediated by overcommitment.

	*T*	*β*	SE	*p*	LLCI	ULCI
Total effect ERI ratio – Perceived Stress	15.2975	0.7902	0.0517	0.000	0.6889	0.8916
Direct effect ERI ratio – Perceived Stress	9.2437	0.5216	0.0564	0.000	0.4109	0.6323
Indirect effect ERI ratio – Overcommitment – Perceived Stress		0.2686	0.0302		0.2135	0.3314

*N* = 1.269. LLCI, lower limit confidence interval; ULCI, upper limit confidence interval.

For hypothesis 3b, using work engagement as the dependent variable, no mediating effect of overcommitment was found (n.s.). Therefore, no further results will be reported.

#### Moderated mediation analyses

Two separate moderated mediations were conducted to test hypotheses 4a and 4b with resilience as the moderator on path a, and perceived stress and work engagement as the dependent variables.

For hypothesis 4a, resilience was found to moderate the effect of ERI-ratio and perceived stress (*β* = 1.25, *SE* = 0.54, *t* = 2.30, *p* = 0.02). Higher overcommitment was associated with higher perceived stress (*β* = 0.52, SE = 0.06, *t* = 9.24, *p* < 0.001).

The overall moderated mediation model was supported with the index of moderated mediation = 0.4 (95% *CI* [0.01, 0.07]).

The conditional indirect effect was the strongest in individuals showing high resilience (*β* = 0.29, *SE* = 0.03, 95% *CI* [0.22, 0.36]) and the weakest in individuals showing low resilience (*β* = 0.23, *SE* = 0.27, 95% *CI* [0.18, 0.29]).

**Figure 2:**
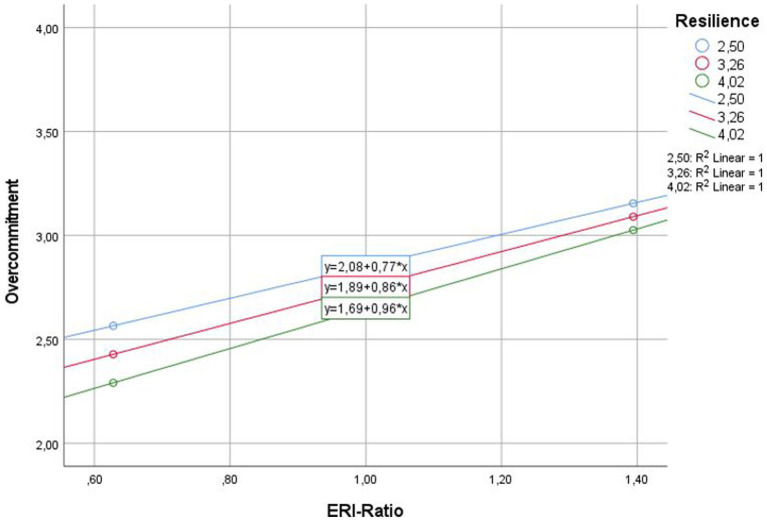
Illustration of Moderated Mediation. *Note*. Overcommitment predicted by ERI-ratio moderated by resilience.

Tests of simple slopes (i.e., conditional effects on path a) found a weaker association between ERI ratio and overcommitment for those with low resilience (*β* = 0.77, *SE* = 0.06, *t* = 12.64, *p* < 0.001) relative to those with high resilience (*β* = 0.96, *SE* = 0.06, *t* = 15.01, *p* < 0.001). PhD students with higher resilience and higher ERI ratio had a higher overcommitment than those with low resilience (Figure 2).

Regarding the dependent variable work engagement, no moderated mediation effect was found (n.s.). Therefore, no further results will be presented.

#### Additional analyses to test lagged effects

To counteract the possible risk of over- or underestimation of possible longitudinal effects in our proposed mediation model (Maxwell and Cole, 2007), we extended our study by a second measurement point. This way, we temporarily separated our predictor variable from the mediator and outcome variables. By doing so, we not only reduced common method bias (Podsakoff et al., 2003), but also were able to test our proposed research model over a period of six weeks. The mediation analyses showed that overcommitment mediated the relationship between ERI and perceived stress over two measurement points (*β* = 0.1782, *SE* = 0.0315, 95% *CI* [0.12; 0.25]) (3a). For the moderated mediation analyses (4a) we found no significant effect over two measurement points (n.s.).

## Discussion

The present study is one of the first to examine the original ERI model to explain well-being in PhD students, incorporating new research findings regarding a possible mediating effect of the negative coping pattern overcommitment. Thus, the relationship between ERI and perceived stress as well as work engagement, via overcommitment, was investigated. In addition, resilience was examined as a potential protective factor between ERI and overcommitment and therefore included as a moderator in the research model. Overall, 1,275 PhD students from across Germany participated in this study.

### Summary of findings and theoretical implications

Before looking at the results of the main analyses, the average ERI ratio of 1.01 for the present sample should be discussed. As explained earlier, an ERI ratio > 1 indicates unfavorable working conditions with high effort and low reward (Siegrist, 2002). Even though an average ERI ratio of 1.01 does not indicate a high imbalance toward efforts, conclusions should not be drawn too quickly. First, it can be assumed that PhD students naturally make great efforts and investments during their doctoral studies, showing high intrinsic motivation (Guerin et al., 2015; Sverdlik et al., 2018). Second, Lehr et al. (2009) found an ERI ratio of 0.49 for healthy teachers and an ERI ratio of 1.03 for teachers with clinical depression, revealing a strong link between an unfavorable ERI and mental disorders. This is supported by Lehr et al. (2010), who propose an alternative cut-off value of 0.72 for the ERI ratio, implying that “a positive imbalance might be essential for healthy working conditions” (p. 258). Lastly, it should be noted that the ERI ratio of 1.01 represents the average value for the population used in this study. However, 590 PhD students showed an ERI ratio of 1.00 or more, with 2.79 being the highest value. Consequently, generally high burdens of PhD students should be acknowledged due to the ERI ratio found in this study.

Looking at the results of hypotheses 1a, 1b, and 1c, incorporating the single scales of the ERI questionnaire, most of the hypotheses could be confirmed with respect to the two outcome variables perceived stress and work engagement. Thus, high effort, low reward, and high overcommitment were expected to be positively associated with perceived stress and negatively associated with work engagement. Regarding the outcome variable perceived stress, as predicted, reward showed a significant negative relationship with perceived stress (H1b), while effort and overcommitment were positively associated with perceived stress (H1c). With respect to the outcome variable work engagement, as expected, there was a positive association between reward and work engagement (H1b), whereas overcommitment was negatively associated with work engagement (H1c). Contrasting hypothesis 1a, effort showed a positive relationship with work engagement.

This positive association between effort and work engagement is noteworthy (H1a), showing that, contrary to assumptions, increasing effort in the doctorate was associated with higher work engagement. This illustrates that for doctoral students, a high level of effort is not necessarily perceived as burdensome but can rather promote work engagement. This is supported by Crawford et al. (2010), showing that while there was a positive relationship between demands and burnout, the relationship was conditioned by the nature of the demands. For example, demands perceived as challenges were positively associated with engagement, whereas demands perceived as barriers were negatively associated with engagement. This seems reasonable as PhD students often show great intrinsic motivation and enthusiasm for their doctoral studies and are therefore willing to invest a lot of time and effort in their doctorate (Guerin et al., 2015; Sverdlik et al., 2018). Thus, not the completion of the dissertation, but rather personal development, intellectual growth, and contribution to society may be the goal for many PhD students (Pretorius and Macaulay, 2021). Furthermore, the results show that the subscale reward exerts the biggest effect on work engagement. This is in line with research showing that work-related resources, such as feedback, social support from peers and leaders as well as development and opportunities for learning are positively related to work engagement (Bakker and Demerouti, 2008). Thus, rewards in the doctorate seem to influence work engagement more than efforts, further reinforcing the assumption of the influence of intrinsic motivation on the doctoral student experience.

Furthermore, hypothesis 2, assuming ERI ratio is positively associated with perceived stress and negatively associated with work engagement, exceeding the effect sizes produced by the single scales, could only be partially confirmed for both outcome variables. While a positive relationship between ERI ratio and perceived stress as well as a negative relationship between ERI ratio and work engagement was found as hypothesized, the models using the three single scales showed higher goodness-of-fit measures for both outcome variables rather than the model including ERI ratio only. In general, the found relationships are supported by other studies (e.g., Waszkowska et al., 2017; Ge et al., 2021; Kamal et al., 2022). However, further investigations need to take place in regard to the outcome variable work engagement as Wang et al. (2017) could also show positive correlations of ERI ratio with work engagement (i.e., vigor, dedication, and absorption).

Moreover, the results of the mediation analysis, including perceived stress as the outcome variable, showed a significant partial mediation between ERI ratio and perceived stress with overcommitment acting as the mediator (H3a). Thus, this study supports current research underlying the belief that overcommitment acts as a mediator instead of a moderator in the ERI model (Theorell, 2017; Hinsch et al., 2019; Hodge et al., 2020; Vilser, 2021). This reinforces the discussion whether overcommitment is a stable personality trait, incorporating elements of the type A behavior pattern (TABP) as originally postulated by Siegrist (1996), or rather a reactive behavior that changes over time depending on the perceived ERI ratio at work. TABP can be described as aggressive, ambitious, and competitive behavior as individuals readily perceive their environment as threatening (Matschinger et al., 1986). Further, a *trait* is defined as a personality pattern that is relatively stable over time and therefore recurs in similar situations, while the term *state* refers to behavior and thoughts that change over time depending on the specific situation the individual is in (Schmitt and Blum, 2020). Acting as a mediator instead of moderator suggests that in the context of PhD students, overcommitment might be modifiable. Thus, if a doctoral student were to experience a better ERI ratio, through, e.g., a change in supervision or the overcoming obstacles, the individual level of overcommitment could change accordingly. The assumption of overcommitment acting as a mediator instead of moderator is further supported by du Prel et al. (2018) showing that changes in external ERI (work-related stress) were associated with a change in overcommitment over time.

Further, a new line of research that compares the overcommitment variable from the ERI model with the psychological detachment variable from the stressor-detachment model underpins this assumption (Kunz et al., 2021). The proximity to the stressor-detachment model becomes apparent when looking at the subscale overcommitment in the ERI questionnaire, which addresses, among other things, switching off from doctoral studies during leisure time. Thus, the subscale overcommitment includes items such as “As soon as I get up in the morning I start thinking about problems related to my PhD” or “I can easily relax and switch off from my PhD.” Interestingly, the question of whether detachment acts as a mediator or a moderator is also debated in the context of the stressor-detachment model, with growing research supporting the former (Sonnentag and Fritz, 2015; Mette et al., 2018; Schulz et al., 2019; Clauss et al., 2020). Thus, psychological detachment was found to mediate the relationship between job demands and well-being indicators, such as exhaustion, fatigue at work, and the need for recovery (Sonnentag and Fritz, 2015). In addition, the temporal sequence of variables was theoretically supported, suggesting that the presence of workplace stressors influences the degree of psychological detachment, which in turn influences stress perception and well-being (Sonnentag and Fritz, 2015).

Regarding the outcome variable work engagement, no mediating effect of overcommitment was found (H3b). By including work engagement as an outcome variable, this study aimed to clarify the role of work engagement in the ERI model, as previous research showed ambivalent results (Kinnunen et al., 2008; Wolter et al., 2021). First, a negative relationship between the ERI ratio and work engagement was found in this study. This is in line with results from Wolter et al. (2021), while Kinnunen et al. (2008) only found a negative association between ERI and two subscales of work engagement, namely vigor and dedication. Furthermore, no mediating effect of overcommitment was detected, even when the three subscales were entered independently into the mediation model in exploratory analyses in this study. This is surprising as the distinctiveness of the construct’s work engagement and overcommitment was confirmed using a CFA. Consequently, the role of work engagement in the ERI model and, in particular, the relationship of work engagement and overcommitment should be further explored in light of the persistent ambivalent findings.

After including resilience as a moderator on path a (the relation of ERI ratio and overcommitment) in the model, a moderated mediation was found for the outcome variable perceived stress (H4a). Surprisingly, resilience moderated the relationship between ERI ratio, via overcommitment, on perceived stress, by further strengthening the association of ERI ratio and overcommitment as the ERI quotient increased. Thus, it appeared that resilience in addition to the perceived ERI was allowing PhD students to endure the adverse circumstances even better, while at the same time, the harmful coping strategy of overcommitment was applied even more. Consequently, the assumption of a protective factor of resilience could not be confirmed for the outcome variable perceived stress (H4a). No moderated mediation was found for the outcome variable work engagement (H4b).

But how can the amplified relationship between ERI ratio and overcommitment due to resilience be explained? First, it could be assumed that resilient individuals temporarily tolerate stressful situations, in this case, a higher ERI during their doctorate, more than their less resilient peers. Because their goal is to best adapt to the adverse environment, more resilient individuals may be more willing to adapt even harmful coping strategies, such as overcommitment, along with beneficial coping strategies to compensate for the perceived imbalance. The challenge of successfully completing the doctorate even under unfavorable conditions can supposedly be mastered with this additional invested time and effort. In the long run, however, the excessive demands might have serious negative consequences for their well-being. The assumption that resilient PhD students only adapt to the adverse environment during the limited time of the doctorate is supported by Chmitorz et al. (2018, p. 1), describing resilience as the “phenomenon that many people maintain mental health or only temporally become mentally ill despite significant adversity.”

This is in line with the second explanatory approach. Even though the results of the study suggest that resilient individuals generally have lower overcommitment scores in the presence of a low ERI, this effect appears to reverse as the ERI ratio increases. Thus, resilience itself did not appear to be a protective factor, especially for high ERI ratios. Therefore, rather than focusing on resilience as a function or behavioral outcome (outcome perspective, e.g., Harvey and Delfabbro, 2004), single aspects of resilience like optimism or specific adaptive coping strategies included in the resilience construct, such as mindfulness, relaxation, and self-reflection, might be crucial to successfully reduce overcommitment (Heckenberg et al., 2019). In addition, Child and Medvedev (2023) postulated that a combination of short-term goal-oriented methods such as stress management, and long-lasting characteristics such as self-efficacy could be beneficial for strengthening resilience in the long term. As only a few studies have investigated adaptive coping strategies to reduce overcommitment, further research is needed on this topic, looking at resilience not only from an outcome, but also from a trait and process perspective, as well as the different elements of resilience.

Lastly, Williams et al. (2018) assume that resilience is more likely to buffer short-term stressful events, such as sexual assault, death, or any other traumatic event. In contrast, an ERI in the context of a doctoral degree is experienced over a longer period and rather unconsciously endured. Schetter and Dolbier (2011, p. 638) suggest that “[…] [f]or resilience to be relevant, a threat, challenge or loss (i.e., a stressor) must be of large enough magnitude to disrupt functioning for […] [an individual].” Thus, an ongoing ERI that slowly evolves over time may not be considered disruptive enough by PhD students for resilience to be triggered as a buffer. Rather, this study supports the notion that resilience appears to amplify the negative effect of chronic stress, such as an ERI at work, as individuals attempt to successfully navigate the negative imbalance they experience (Schetter and Dolbier, 2011). Resilience might therefore only have a cushioning effect within a reasonable ERI ratio, while having a negative effect as challenges increase.

Looking at the results of our additional analysis, our proposed mediation model, namely overcommitment being a mediator in the relationship between ERI and perceived stress, was supported over time. These findings are in line with Vilser (2021), who found overcommitment to mediate the relationship between ERI and perceived stress in judges over time. Our results are particularly relevant as they demonstrate that, in the context of ERI, the use of the harmful coping strategy overcommitment predicts actual changes in the well-being of PhD students over a period of time. Furthermore, with overcommitment as a mediator between ERI and perceived stress, our results underline the assumption that overcommitment is a crucial mechanism in the functioning of ERI and well-being.

Finally, including the control variables in the analyses, this study supports the assumption that the COVID-19 pandemic negatively influenced the overall doctoral experience of PhD students. This is reinforced by Anders et al. (2022) who demonstrated that contact restrictions, interruptions in research projects due to closed laboratories and libraries as well as canceled conferences due to the pandemic were a major burden for PhD students. Age, on the other hand, was found to positively influence the doctoral experience supporting the assumption that as age increases, so do resources, which in turn contribute to better coping with work stressors (Kim and Kang, 2017). Furthermore, the model of selection, optimization, and compensation (SOC) might be an explanation for the beneficial effect of age in PhD students. According to Baltes and Baltes (1990), as we age, while getting older we maximize gains and minimize losses associated through selection, optimization, and compensation strategies, meaning we are dealing more successfully with challenges over our lifespan. Therefore, older PhD students might be able to cope better with adverse circumstances. Also, it should be noted that far more women (64%) than men participated in this study, which may have several reasons. For instance, studies such as Sverdlik et al. (2023) found higher stress levels among female doctoral students, suggesting that women may be more susceptible to stress and anxiety than male PhD students. This may increase interest in understanding and addressing these challenges through engagement in research on well-being – as this topic was explicitly stated in the study’s invitation. Also, several studies have consistently shown that women report higher stress levels compared to men across diverse samples (i.e., Misra and McKean, 2000; Matud, 2004; Graves et al., 2021). This could mean that women are more inclined to take part in a survey on the subject of stress, leading to a greater number of female respondents.

### Practical implications

Although the protective role of resilience could not be demonstrated, this study provides important implications for practice, both for doctoral students and institutions (e.g., universities, non-university research institutes, funding organizations for promotion, and supervisors).

First, the results of this study support the assumption that the negative coping pattern overcommitment is not a rigid personality trait, but can rather be modified. Personal development workshops might help PhD students acquire positive coping strategies and successfully overcome self-sabotaging behavior, such as overcommitment. There, the goal should not only be to teach general project management skills such as time management or goal setting, but also to identify and change harmful behaviors and beliefs (Kearns et al., 2008). Furthermore, open conversations about the experienced imbalance with supervisors or relevant contacts could help to make the stress perception visible. High time pressure, working overtime, frequent interruptions, and fears about the future can thus be addressed in this way, helping supervisors and PhD students to openly communicate their needs and gradually align their working styles (Sverdlik et al., 2018). Also, as this study strengthens the conceptual proximity of overcommitment to psychological detachment, exercising and spending time with others in leisure time as well as switching off over a longer period of time, for example by taking vacations, is recommended (e.g., Martinez et al., 2013; Sonnentag and Fritz, 2015). Finally, critical self-reflection and questioning of supposedly helpful coping strategies could help doctoral students seek timely support and improve the overall doctoral experience, especially as the experienced ERI increases (Heckenberg et al., 2019; Ang et al., 2022).

At the same time, this is a call to supervisors and institutions (e.g., universities, funding organizations). This study suggests that resilience in PhD students seems to be rather harmful than helpful. However, the doctoral experience is fundamentally shaped by supervisors and institutions, which therefore need to take on a stronger role as active companions and supporters in the doctoral process (e.g., Kearns et al., 2008; Beasy et al., 2021). Thus, as doctoral students are likely to show high intrinsic motivation and a propensity for overcommitment (Sverdlik et al., 2018; Hamilton, 2019), supervisors should keep an eye on the workload of PhD students, such as additional teaching assignments and research projects. Additionally, supervisors are encouraged to model a healthy work-life balance to motivate PhD students to follow their lead as proposed by the social learning theory (Bandura, 1977). Since the results suggest that rewards are critical to both work engagement and perceived stress, rewards should be more strongly encouraged. While salary is often unchangeable due to external funding programs or scholarships, recognition, appreciation, and development depend on the supervisor and thus can be influenced. This includes for example the celebration of successes, regular communication, feedback, and goal setting as well as opportunities for PhD students to attend training or participate in conferences. Also, establishing long-term contracts to foster job security or mentoring discussions about future development possibilities are conceivable ways to increase the reward component (Sverdlik et al., 2018; de Vries, 2020). Accordingly, supervisors are encouraged to envision themselves as leaders and mentors and should therefore be trained in employee-centered or transformational leadership styles to enhance doctoral students’ well-being and job satisfaction. Passivity, *laissez-faire* leadership, or inadequate leadership skills, on the contrary, can be detrimental to the health and well-being of PhD students (Bauer and Kuschel, 2020; Anders et al., 2022).

At the institutional level, universities, and foundations should help to promote resources for PhD students by creating networking and exchange opportunities with peers and scholars (e.g., through peer mentoring programs, group coaching, or networking events). Like this, the integration of doctoral students into the scientific community could be improved (Stubb et al., 2011). Also, job security should be increased at the institutional level through long-term contracts, higher salaries, and continuing training opportunities (de Vries, 2020; Beasy et al., 2021). Furthermore, supportive infrastructures, such as career counseling, psychological advisory services, and bureaucratic support, are needed to help PhD students through difficult times in their doctorate (Anders et al., 2022). This is supported by a comprehensive error culture that normalizes setbacks in science and makes room for mistakes and experimentation, as doctoral students learn how to deal better with failures (Anders et al., 2022). Lastly, the digitalization and hygiene concepts of universities should be further advanced to ensure uninterrupted research processes during the pandemic (Anders et al., 2022).

It is assumed that by implementing these practical implications, the overall experience of doctoral students can be improved while reducing the negative health consequences of ERI and overcommitment.

### Limitations and implications for future research

For this study, there are both limitations and implications for future research. First, even though this study supports the assumption that overcommitment acts as a mediator instead of a moderator, the question arises whether both mechanisms are plausible as proposed in the stressor-detachment model. According to state/trait models, this would mean that the individual trait overcommitment is triggered more or less depending on the specific situation (Schmitt and Blum, 2020). Future research could therefore focus on looking at overcommitment from a state/trait perspective, thus exploring a possible interaction as well as mediation at the same time.

Furthermore, it should be considered whether hypothesis 2, focusing on exceeding the effect sizes of ERI ratio on the effect sizes produced by the single scales, could also be measured using different statistical methods as “Siegrist does not specify whether the interaction hypothesis refers to additive main effects or to a synergistic effect […] most studies have tested for the interaction hypothesis on a variable level using regression analysis.” (Lau, 2008, p. 2). However, also other methods could be used, such as multivariate regression analysis (testing if the combined measure has a significant impact, e.g., on health), a mediation analysis (testing if the mediation effect is bigger than individual effects), or a moderation (testing if specific variables influence the relationship between individual measures).

Another limitation concerns the investigated moderator resilience. As described above, different conceptualizations of resilience exist, namely trait-, process- and outcome-oriented viewpoints (Hu et al., 2015; Joyce et al., 2018). This study adopted an outcome-oriented approach as it was assumed that resilience acts as the result of successfully applied coping strategies. However, the results of this study showed no general protective effect of resilience, as resilience even strengthened the relationship between ERI ratio and overcommitment, especially with an increasingly unfavorable ERI ratio. This suggests that not resilience as an outcome, but rather individual components of resilience, such as problem-solving skills or optimism (Ang et al., 2022), could act as potential protective factors. This is also in line with research on the stressor-detachment model, which shows that individuals who have more personal and work-related resources, such as self-efficacy and social support, are better able to wind down after work than individuals who lack these resources. Furthermore, Soucek et al. (2016) stress that besides resilience, other personal resources such as optimism, self-efficacy, and hope are considered beneficial in the work context, as part of the theoretical construct of psychological capital (Luthans et al., 2015). It is therefore recommended for future research to concentrate on specific components of resilience as well as related constructs and thus systematically take possible moderators into account to better understand which coping styles are beneficial and which are rather detrimental.

As a fourth limitation, it should be noted that the study is not a laboratory study under controlled conditions, but a self-report questionnaire study. Possible biases due to confounding variables in the individual environment of the doctoral students when filling out the questionnaire or common method bias (Podsakoff et al., 2003) can therefore not be excluded. However, this format supported the wide range of this study across Germany and therefore outweighs possible methodological biases. Nevertheless, future research should investigate the ERI model using more objective outcome criteria such as work performance or physiological well-being, measured for example through blood pressure or cortisol levels. Furthermore, an experimental design could be applied using vignettes to manipulate the experienced ERI of participants. In these vignettes, supervisor behavior (e.g., transformational vs. non-transformational leadership) or frame conditions of the doctorate could be manipulated, for example by describing different levels of efforts (e.g., workload, need for overtime) and/or rewards (e.g., convention trip, permanent employment contract).

Additionally, further research is needed to investigate the role of work engagement and its subscales as well as other positive outcomes such as job satisfaction in the ERI model, as no clear picture has emerged yet from previous research (Wolter et al., 2021) nor this study.

Lastly, there is evidence that women experience more stress during their doctorate (Kurtz-Costes et al., 2006). The reasons for this are greater challenges of women due to additional responsibilities and conflicts in their personal lives as well as greater difficulty in obtaining equal chances and resources in the scientific environment as men (Hazell et al., 2020). However, in this study, no support for a gender effect regarding the outcome variable perceived stress was found. Accordingly, further research should examine possible gender effects, especially with respect to doctoral students in Germany.

## Conclusion

A doctorate is associated with numerous challenges for many doctoral students. Besides financial insecurities, self-doubts, and time pressure, an experienced imbalance between effort and reward seems to play a major role in the well-being of PhD students. This study is one of the first to examine the original ERI model in PhD students, incorporating new research findings regarding a possible mediating effect of the negative coping pattern overcommitment. Thus, the relationship between ERI and perceived stress as well as work engagement, via overcommitment, was investigated.

It was found that overcommitment mediated the relationship between ERI and perceived stress, while no mediation was found for work engagement. In addition, resilience was examined as a potential protective factor between ERI and overcommitment. Here it could be shown that overcommitment increases with an increasing ERI ratio, while the relationship is moderated by resilience. Less resilient individuals consistently showed higher overcommitment scores than more resilient individuals, but contrary to expectations, resilience seemed to reinforce the relationship between ERI ratio and overcommitment with an increased ERI ratio. Thus, it appeared that with increasing ERI, resilient PhD students were even more likely to adopt the harmful coping strategy of overcommitment. Therefore, the expected buffering effect of resilience could not be supported. The authors discussed that resilient individuals could temporarily tolerate stressful situations because their goal is to best adapt to the adverse environment. In the long term, however, the excessive demands have serious negative consequences. Therefore, practical implications should be considered such as mindfulness interventions, personal development workshops, open conversations about the perceived stress as well as supervisor training and changes on the institutional level (e.g., regarding the salary).

To sum it up, this study adds value to empirical research not only by providing a deeper understanding of the ERI model and its mechanisms in PhD students, but also by triggering the investigation of possible protective factors of the negative coping pattern overcommitment.

## Data availability statement

The original contributions presented in the study are included in the article, further inquiries can be directed to the corresponding author.

## Ethics statement

Our study strictly adhered to the Ethical Guidelines of the German Association of Psychologists (DGPs), the American Psychological Association (APA), and the Department of Psychology at Ludwig-Maximilians-University Munich (LMU). As it didn’t involve sensitive personal data, impact vulnerable groups, or pose risks to participants, ethical approval wasn’t necessary according to national and institutional guidelines. The study employed an anonymous questionnaire, without collecting any identifying information. All participants provided written informed consent, were comprehensively briefed on confidentiality, and had the freedom to withdraw without giving explanation.

## Author contributions

MV: Conceptualization, Data curation, Methodology, Project administration, Supervision, Writing – original draft, Writing – review & editing. SG: Conceptualization, Data curation, Formal analysis, Investigation, Methodology, Writing – original draft, Writing – review & editing. IM: Conceptualization, Methodology, Supervision, Writing – review & editing.
